# Effect of Pelvic Bone Marrow Sparing Intensity Modulated Radiation Therapy on Acute Hematologic Toxicity in Rectal Cancer Patients Undergoing Chemo-Radiotherapy

**DOI:** 10.3389/fonc.2021.646211

**Published:** 2021-04-22

**Authors:** Wei Huang, Jun Dang, Ying Li, Hai-xia Cui, Wen-li Lu, Qing-feng Jiang

**Affiliations:** Department of Oncology, The First Affiliated Hospital of Chongqing Medical University, Chongqing, China

**Keywords:** rectal cancer, pelvic bone marrow, IMRT, acute hematologic toxicity, chemo-radiotherapy

## Abstract

**Background:**

While chemo-radiotherapy improves local control in patients with locally advanced rectal cancer, it can also increase acute hematological toxicity (HT), which leads to poor outcomes. Patients receiving bone marrow radiation have been shown to develop acute HT. However, the safety and efficacy of bone marrow sparing is undetermined. The aim of our study was to explore the feasible dosimetric constraints for pelvic bone marrow (PBM) that can be widely used in rectal cancer patients undergoing chemo-radiotherapy.

**Methods:**

112 rectal cancer patients were selected and divided into the PBM sparing IMRT group (60 cases) and the non-PBM sparing IMRT group (52 cases). All patients underwent pelvic radiotherapy with concurrent capecitabine-based chemotherapy. The PBM dosimetric constraints in the PBM sparing IMRT group were set to:V_10_ ≤ 85%, V_20_ ≤ 65% and V_30_ ≤ 45%. An independent sample t test was applied for the dose-volume parameters, and Chi-squared analysis was applied for clinical parameters and adverse events.

**Results:**

The radiation dose to PBM (V_5_~V_45_, D_mean_, *P*<0.05), PBM sub-regions (V_10_~V_35_, D_mean_, *P*<0.05) and both femoral heads (V_5_~V_40_, D_mean_, *P*<0.05) decreased significantly in the PBM sparing IMRT group compared with that of the non-PBM sparing IMRT group (*P*<0.05). There was no significant difference in any dose-volume parameters of the bladder and small bowel in either groups, and none in the planning target volume (PTV) dose homogeneity and conformity (*P*>0.05). For acute HT observation, the incidence of grade 3 acute HT (χ^2^ = 7.094, *P*=0.008) was significantly reduced in patients treated with PBM sparing IMRT compared with patients treated with non-PBM sparing IMRT. There was no statistical difference in the incidence of vomiting, diarrhea, fatigue, anorexia, nausea, hand-foot syndrome, cystitis, perianal pain and perianal dermatitis in patients of both groups (*P >*0.05).

**Conclusions:**

Applying PBM dosimetric constraints (V_10_ ≤ 85%, V_20_ ≤ 65% and V_30_ ≤ 45%) can significantly reduce the radiation dose to PBM. The patients treated with PBM sparing IMRT had a lower incidence of acute HT compared with those treated with non-PBM sparing IMRT. Applying the PBM dosimetric constraints proposed by our study can benefits the patients with rectal cancer undergoing capecitabine-based chemo-radiotherapy.

## Introduction

Rectal cancer is one of the most common malignancies in the world (1). Chemo-radiotherapy (CRT) has been widely used in the treatment of rectal cancer. It improves local control in patients with locally advanced rectal cancer (2–4). However, CRT also increases acute hematological toxicity (HT) (5–8). Serious HT could reduce the intensity and benefit of chemotherapy or even cause treatment interruptions (9–11), which has been associated with poor outcomes. Reducing HT is, therefore, an important approach for improving the therapeutic ratio of CRT.

Both radiation and chemotherapy are myelosuppressive. Chemotherapy suppresses hematopoiesis in active bone marrow (BM) throughout the body. Meanwhile, radiotherapy causes BM stromal damage and apoptosis of active BM stem cells (12, 13) within the irradiation fields. As the pelvic bone marrow (PBM) accounts for nearly 40% of total BM (14), the local regional radiation effects exacerbates the acute HT, particularly in patients undergoing CRT (15, 16). Clinical studies have shown that the extent of radiation-induced BM injury depends on both radiation dose and volume of PBM irradiated (17–20), suggesting that reducing the PBM radiation dose could reduce the acute HT.

To efficiently reduce the radiation dose to PBM, a series of studies have been performed over the past ten years to investigate the correlation of PBM dose-volume parameters with the incidence of acute HT. Many studies have found that the incidence of acute HT was significantly associated with PBM V_10_ (<90% or <95% was recommended) (15, 21), V_20_ (<76% was recommended) (21) and the average dose (<22.5Gy or <25Gy was recommended) (22) in patients with cervical cancer or anal cancer. However, the delineation of the target for rectal cancer is quite different from that of cervical cancer or anal cancer, which leads to a significant difference in dose distributions of PBM in patients with rectal cancer. Therefore, it is still unknown whether those PBM dosimetric constraints are suitable for patients with rectal cancer. As for rectal cancer, few studies have been found particularly on rectal cancer patients undergoing CRT. To our knowledge, only two retrospective studies demonstrated a correlation between high-dose parameters (lumbosacral spine BM V_40_ and V_45_) in the PBM sub-region and acute HT. However, their recommended lumbosacral spine BM V_40_ <60% (10) and V_45_ <51% (9) were relatively loose compared with that of our clinical practice, which we will discuss later in this study. Therefore, it is necessary to explore a more practical guideline for PBM dosimetric constraints for rectal cancer patients.

Our study aimed to investigate feasible dosimetric constraints of PBM for rectal cancer patients during IMRT plan optimization, so that the radiation dose to the PBM could be reduced efficiently without either sacrificing the dose coverage of the target volume or increasing the radiation dose to other organs at risk (OARs), thus reducing the incidence of acute HT for rectal cancer patients undergoing CRT eventually.

## Methods and Materials

### Clinical Data

All patients 1) had a KPS score of ≥70; 2) had histologically confirmed rectal adenocarcinoma located <12 cm from anal verge; 3) had adequate function of major organs (including cardiac, hepatic, and renal functions);4) had adequate bone marrow function (hemoglobin > 10 g/dL; absolute neutrophil count (ANC) ≥ 1800/μL; platelet count ≥ 100,000/μL; leukocyte count ≥ 3500/μL); 5) underwent radiotherapy and concurrent capecitabine based chemotherapy; 6) completed the scheduled radiotherapy. Patients were excluded if they had central nervous system disorders, or psychological disability, or a second primary tumor other than nonmelanoma skin cancer or *in situ* cervical carcinoma. Patients were also excluded if they had serious, uncontrolled infections.

We prospectively analyzed 112 rectal cancer patients treated with concurrent chemotherapy at our institution between 2012 and 2019. Fifty-two rectal cancer patients (between January 2012 and June 2016) were included in the non-pelvic bone marrow sparing IMRT (non-PBM sparing IMRT) group, all of whom were treated with non-PBM sparing IMRT when undergoing radiotherapy; another 60 rectal cancer patients (between June 2016 and October 2019) were enrolled in the pelvic bone marrow sparing IMRT (PBM sparing-IMRT) group. They were treated with PBM sparing IMRT when undergoing radiotherapy. All patients underwent concurrent capecitabine based radio-chemotherapy. Capecitabine was given at a dose of 825 mg/m^2^ twice daily from Monday to Friday throughout the entire course of IMRT. Tumor histology, stage, age, Body Mass Indices (BMI) and comorbidity was collected for all patients. The median value for age, BMI, comorbidities and distance between the tumor and the anus was 60.0 years old, 23.1 kg/m^2^, 1.0 and 5.0 cm, respectively for all patients. There were no statistical differences for clinical factors between the two groups (**Table 1**). The study was approved by our institutional review board. All patients provided written informed consent.

**Table 1:** Chi-squared analysis of clinical factors for PBM sparing IMRT group and non-PBM sparing IMRT group.

Clinical factors	Classification	Number		χ^2^	*P*
		PBM sparing IMRT group	non-PBM sparing IMRT group		
Age	<60	32	28	0.003	0.957
	≥60	28	24		
Sex	Male	40	30	0.957	0.328
	Female	20	22		
Comorbidity*	≤1	40	35	0.005	0.943
	>1	20	17		
BMI (kg/m^2^)	<23	38	26	2.022	0.155
	≥23	22	26		
T classification	2	2	6	2.828	0.093
	3-4	58	46		
N classification	0	15	12	0.056	0.812
	1-2	45	40		
Distance between tumor	<5	23	22	0.183	0.669
and anal verge(cm)	≥5	37	30		

*Comorbidity = the number of chronic diseases diagnosed at the same time when a patient was diagnosed with rectal cancer.

### Treatment Planning

All patients were instructed to drink 500 ml water and empty the rectum one hour prior to the CT scan and daily radiotherapy treatment. All patients were simulated in the supine position with customized immobilization. A pelvic scan using GE LightSpeed RT 4-row CT with a scan layer thickness of 5 mm were performed on all patients. The images were digitally transmitted and 3D reconstructed into the treatment planning system (Varian Eclipse, USA).

**Figure 1:**
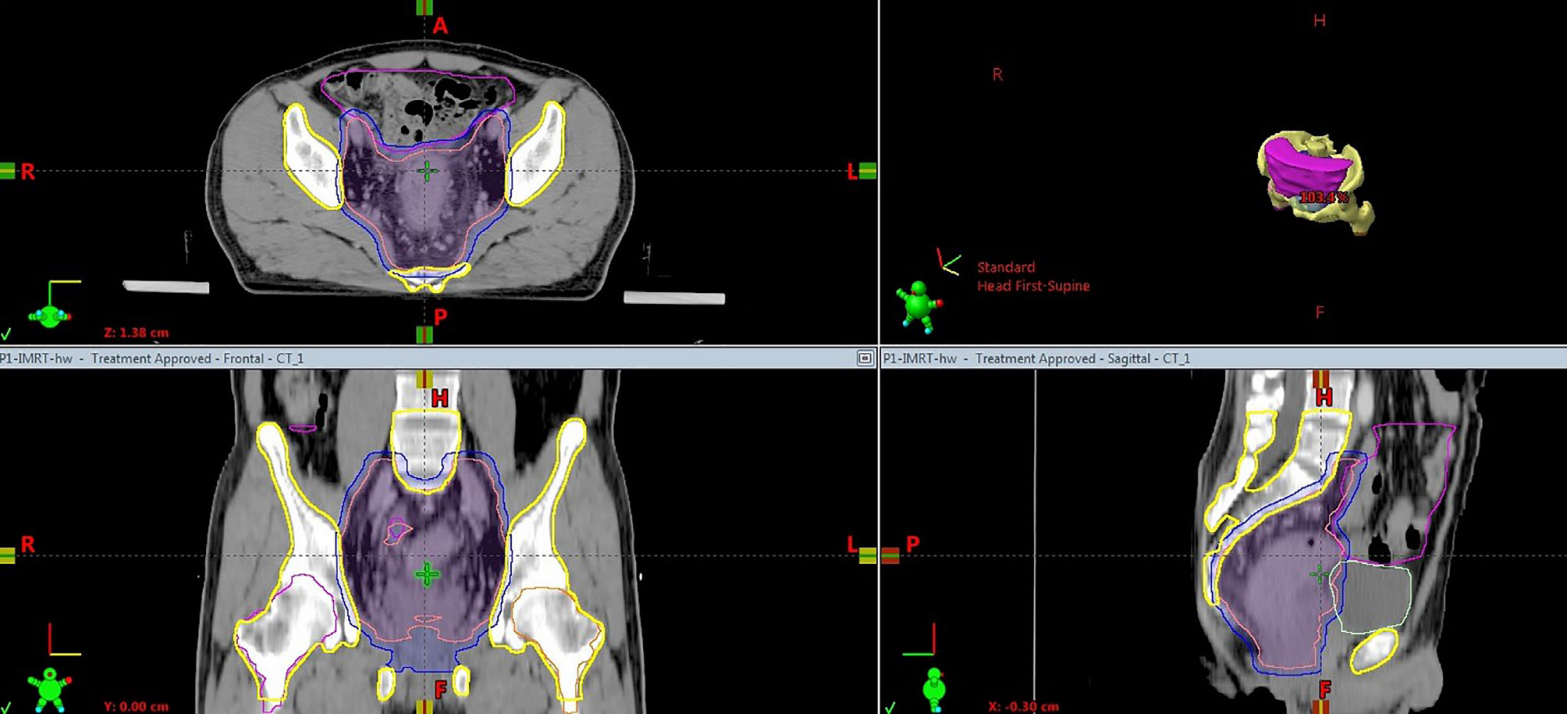
Computed tomography slice showing the delineation of target volume and OARs. Pink solid, clinical target volume; blue solid, planning target volume; yellow, pelvic bone marrow; magenta, small bowel; light green, bladder.

The target was delineated according to the international consensus guidelines (23–25). The bladder, small bowel, femoral heads and PBM were defined as OARs. The small bowel was outlined to include the entire peritoneal potential space of bowel (26). Pelvic bone structures were contoured on the planning CT using bone windows, and the contouring range was defined from the fifth lumbar vertebra to the ischial tuberosities. For further data analysis, the PBM was divided into three sub-regions (lumbosacral spine BM, ilium BM and lower pelvis BM), as described by Mell L K et al. (15). **Figure 1** shows a representative CT slices with delineation of target volume and OARs.

All patients underwent radiotherapy with 6 MV X-rays. Before March 2016 only the fixed-field IMRT technique was applied to patients in the non-PBM sparing IMRT group. The Volumetric Modulated Arc Therapy (VMAT) technique was gradually introduced after March 2016. Since then both the fixed-field IMRT and VMAT techniques were applied to patients in both groups. For fixed-field IMRT plans, 7 coplanar, equally shot, sliding-window fields were generated and applied before optimization. Forty-one patients in the non-PBM sparing IMRT group and 31 in the PBM sparing IMRT group were treated with fixed-field IMRT technique. For VMAT, 2 semi-arc (0~179° and 181~0°) and 1 full arc (179~181°) were applied. Eleven patients in the non-PBM sparing IMRT group and 29 in the PBM sparing IMRT group were treated using the VMAT technique. It should be noted that comparing VMAT with fixed-field IMRT was not a prespecified objective in our study.

The optimization priority in PBM sparing IMRT group was: PTV> bladder, small bowel> femoral heads > PBM, and the corresponding order in the non-PBM sparing IMRT group was: PTV> bladder, small bowel > femoral heads. The PTV prescription doses ranged from 50~50.4 Gy in 25~28 daily fractions, 5 fractions per week. All plans were normalized to cover 95% of the PTV volume with 100% of the prescription dose. The OARs dosimetric constraints for both groups were set as follows: bladder V_50_ (volume receiving≥50Gy) ≤30%; bladder V_40_ ≤60%; small bowel V_45_ ≤195 cc; femoral heads V_45_ ≤5% for both groups. PBM dosimetric constraints for patients in the PBM sparing IMRT group were set as: PBM V_10_≤ 85%, V_20_≤ 65% and V_30_≤ 45%.

### Safety Assessment

All patients treated with chemo-radiation therapy had complete blood counts with differentials weekly during treatment and every 2 weeks after treatment. Anti-emetics and antidiarrheal agents were prescribed when needed.

### Evaluation Parameters

Dose-volume parameters were generated for the PTV and OARs (including small bowel, bladder, femoral heads, PBM and its sub-regions). To evaluate PTV coverage, dose conformity indices (CI), dose homogeneity indices (HI), D_2_ and D_98_ were calculated. The dose-volume parameters for OARs refer to the absolute volume of small bowel and percentage volume of bladder, femoral heads, PBM and its sub-regions which receive doses greater than 5, 10, 15, 20, 25, 30, 35, 40, 45, 50 Gy (V_5_, V_10_, V_15_, V_20_, V_25_, V_30_, V_35_, V_40_, V_45_, V_50_), as well as the mean dose(D_mean_).

Adverse events included acute HT, vomiting, diarrhea, fatigue, anorexia, nausea, hand-foot syndrome, cystitis, perianal pain and perianal dermatitis. Acute HT was counted by the lowest value among the values of white blood cell, absolute neutrophil count, hemoglobin, and platelet within 90 days after the beginning of radiotherapy. Adverse events were graded according to Common Terminology Criteria for Adverse events version 5.0.

### Statistical Analysis

In our study, we hypothesized that by using PBM sparing, we were able to reduce the incidence of Grade ≥ 3 acute HT from 17% to 2%. At least 49 patients in each group were needed to be enrolled in the experiment according to optimal two-stage designs (α = 0.05, β =0.2).

Statistical analysis was performed using SPSS 22.0. Independent sample t test was applied for the dose-volume parameters and all results were demonstrated as $\bar{x}\pm s$. Adverse events in acute phase and clinical data were compared by chi-squared analysis. *P*<0.05 was considered statistically significant.

## Results

### Comparison of Dose-Volume Parameters Between Two Groups

**Figure 2:**
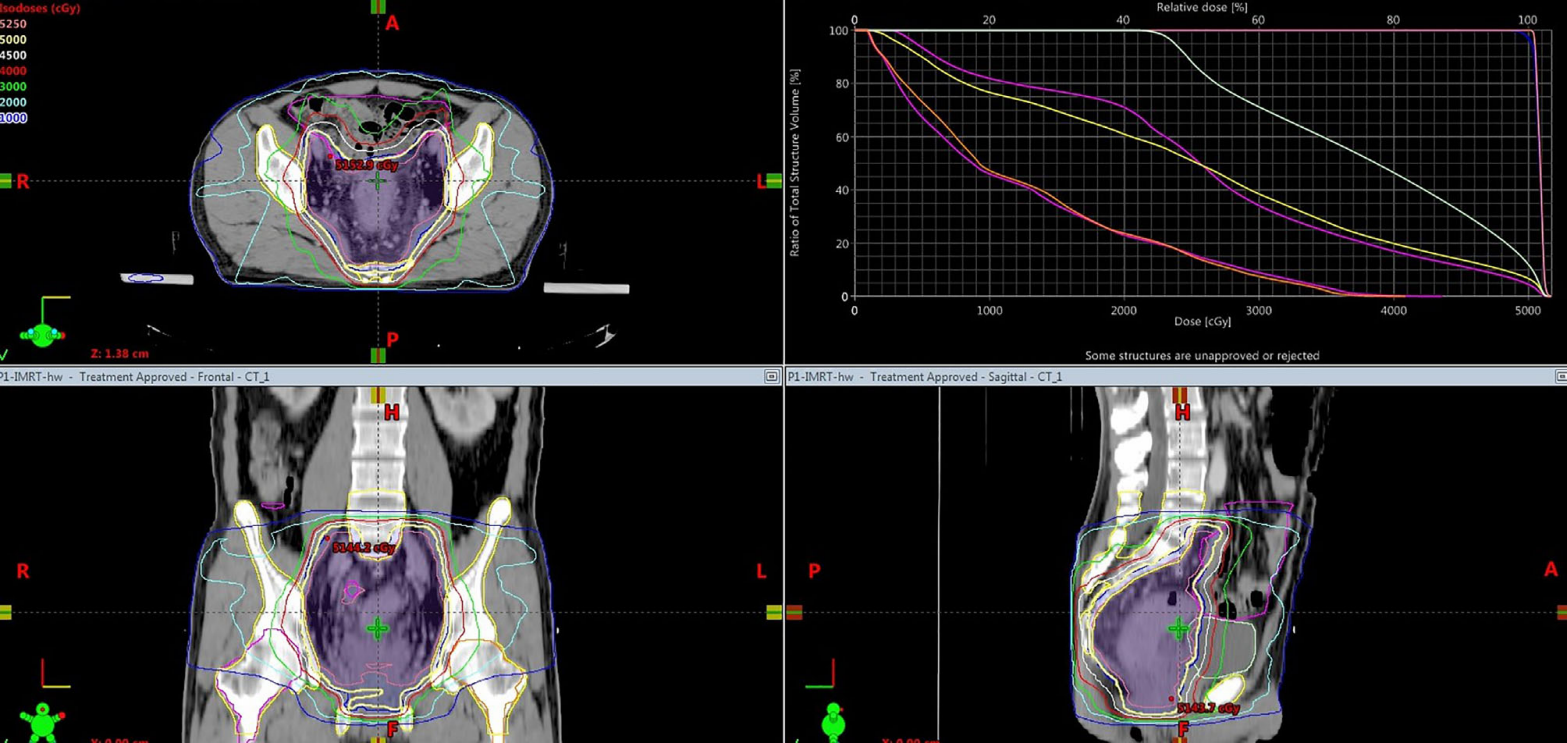
Dose distribution (isodose line) and dose volume histogram of a representative PBM sparing 7 fixed-field IMRT plan. The dose prescription for this case was 50Gy.

**Figure 2** presents a representative PBM sparing IMRT plan. As for dose distribution, both groups generated qualified PTV dose coverage (**Table 2**). The D_95_ of the PTV was 100% in all patients as prescription dose (50~50.4 Gy) was prescribed to encompass 95% of the PTV during IMRT plan optimization. There was no statistical difference in D_2_ or D_98_ of the PTV in PBM sparing IMRT group compared with the non-PBM sparing IMRT group (*P*>0.05). In addition, both groups achieved satisfactory dose homogeneity (HI <0.10) and dose conformity (CI>0.80). The conformity in the PBM sparing IMRT group was slightly higher than that in the non-PBM sparing IMRT group, although without any statistical difference (0.874 ± 0.028 V.S. 0.859 ± 0.054, P=0.128). There was no statistical difference in PTV dose homogeneity (0.0068 ± 0.033 V.S. 0.071 ± 0.025, P =0.662) in both groups.

**Table 2:** Comparison of the PTV dose distribution between the PBM sparing IMRT group and the non-PBM sparing IMRT group.

	D_2_ (Gy)	D_98_ (Gy)	HI*	CI*
PMBS-IMRT Group	52.52 ± 1.80	49.16 ± 0.95	0.068 ± 0.033	0.874 ± 0.028
non-PBM sparing IMRT group	52.54 ± 1.64	49.02 ± 1.55	0.071 ± 0.025	0.859 ± 0.054
*P*	0.993	0.629	0.662	0.128

*HI indicates the dose homogeneity indices, CI represents the dose conformity indices. HI = (D_2_-D_98_)/D_prescription_ *100%,where D_2%_, D_98_, D_prescription_ represent radiation dose delivered to 2%, 98%, and 100% of the PTV, respectively.CI = V_tref_/V_t_ × V_tref/_V_ref_, where V_t_, V_tref,_ V_ref_ are the volume of the PTV,the volume of the PTV covered by the prescription dose and the volume surrounded by the prescription isodose curve.

**Table 3** shows that the radiation dose to PBM (V_5_~V_45_, D_mean_, *P*<0.05), PBM sub-regions (V_10_~V_35_, D_mean_, *P*<0.05) and both femoral heads (V_5_~V_40_, D_mean_, *P*<0.05) decreased significantly in the PBM sparing IMRT group compared with that of the non-PBM sparing IMRT group (*P*<0.05). There was no statistical difference in bladder or small bowel for any dose-volume parameters between those two groups (*P*>0.05). In the meantime, it can be observed from **Table 4** that most of the volume of PBM and its sub-regions that received low dose(except for V_5_ of lumbosacral spine BM) were reduced significantly in the PBM sparing IMRT Group, especially inV_10_~V_35_ and D_mean_(*P*<0.05). Among them, the sparing PBM V_10_ (82.1 ± 6.4 V.S. 91.6 ± 5.6, *P<0.001*), V_20_ (62.4 ± 5.7 V.S. 78.1 ± 8.4, *P<0.001*), V_30_ (40.8 ± 5.3 V.S. 51.8 ± 8.0, *P<0.001*) were particularly reduced in PBM sparing IMRT group, as was the V_40_ (22.8 ± 4.6 V.S. 26.8 ± 6.2, *P<0.001*) of PBM. We did not observe significant differences for V_50_ of PBM, V_40_~V_50_ of the lumbosacral spine BM and ilium BM in both groups (*P*>0.05).

**Table 3:** Independent sample *t* test of OARs’ dose-volume parameters between PBM sparing IMRT group and non-PBM sparing IMRT group $\left(\bar{x}\pm s\right)$.

Dose-volume parameters	Bladder		*P*	Small bowel		*P*	Left femoral head		*P*	Right femoral head		*P*
	PBM sparing IMRT (%)	non-PBM sparing IMRT (%)		PBM sparing IMRT (cc)	non-PBM sparing IMRT (cc)		PBM sparing IMRT (%)	non-PBM sparing IMRT (%)		PBM sparing IMRT (%)	non-PBM sparing IMRT (%)	
*V*_5_	100.0 ± 0.0	100.0 ± 0.0	0.285	1053 ± 535	1056 ± 370	0.968	79.8 ± 15.6	95.0 ± 7.5	<0.001	78.6 ± 17.8	94.7 ± 8.0	<0.001
*V*_10_	100.0 ± 0.2	100.0 ± 0.0	0.354	984 ± 499	995 ± 353	0.896	53.7 ± 20.6	84.9 ± 15.0	<0.001	53.8 ± 21.7	84,.0 ± 16.4	<0.001
*V*_15_	99.9 ± 1.1	99.9 ± 0.5	0.749	907 ± 456	934 ± 336	0.722	37.2 ± 18.5	74.1 ± 19.9	<0.001	36.2 ± 19.7	73.9 ± 20.6	<0.001
*V*_20_	99.1 ± 3.1	99.3 ± 1.8	0.718	806 ± 390	840 ± 314	0.614	23.7 ± 13.7	57.4 ± 21.4	<0.001	23.2 ± 17.2	55.7 ± 20.8	<0.001
*V*_25_	94.4 ± 7.9	96.3 ± 5.6	0.141	663 ± 281	683 ± 265	0.691	12.9 ± 9.3	35.9 ± 17.3	<0.001	12.6 ± 12.1	33.6 ± 17.2	<0.001
*V*_30_	82.2 ± 13.7	86.5 ± 9.8	0.062	503 ± 209	532 ± 215	0.583	6.0 ± 5.8	20.4 ± 12.5	<0.001	5.8 ± 6.1	18.4 ± 12.6	<0.001
*V*_35_	64.2 ± 16.5	67.9 ± 12.3	0.182	372 ± 161	391 ± 176	0.552	2.6 ± 3.4	8.3 ± 5.3	<0.001	2.6 ± 3.5	7.6 ± 6.2	<0.001
*V*_40_	47.9 ± 16.4	49.8 ± 13.4	0.505	268 ± 122	280 ± 142	0.625	1.0 ± 1.7	2.5 ± 2.7	0.001	1.0 ± 2.0	2.5 ± 2.7	0.001
*V*_45_	33.5 ± 14.8	33.0 ± 12.9	0.838	193 ± 98	199 ± 118	0.784	0.3 ± 0.6	0.6 ± 1.1	0.068	0.4 ± 0.9	0.5 ± 0.9	0.358
*V*_50_	16.8 ± 11.7	15.4 ± 10.9	0.523	110 ± 74	104 ± 94	0.688	0.0 ± 0.0	0.1 ± 0.3	0.284	0.0 ± 0.2	0.0 ± 0.1	0.946
*D_mean_*	3932 ± 356	3977 ± 272	0.388	3009 ± 418	3069 ± 432	0.459	1340 ± 393	2146 ± 433	0.001	1318 ± 436	2114 ± 436	<0.001

**Table 4:** Independent sample *t* test of dose-volume parameters for PBM and its sub-regions between PBM sparing IMRT group and non-PBM sparing IMRT group $\left(\bar{x}\pm s\right)$.

Dose-volume parameters	PBM		*P*	Lumbosacral spine BM		*P*	Ilium BM		*P*	Lower pelvic BM		*P*
	PBM sparing IMRT (%)	non-PBM sparing IMRT (%)		PBM sparing IMRT (%)	non-PBM sparing IMRT (%)		PBM sparing IMRT (%)	non-PBM sparing IMRT (%)		PBM sparing IMRT (%)	non-PBM sparing IMRT (%)	
*V*_5_	92.5 ± 5.4	97.2 ± 3.3	<0.001	93.8 ± 8.7	87.4 ± 6.3	0.014	96.2 ± 5.9	98.3 ± 3.6	0.023	88.3 ± 10.6	96.4 ± 5.0	<0.001
*V*_10_	82.1 ± 6.4	91.6 ± 5.6	<0.001	89.2 ± 11.2	95.0 ± 8.5	0.002	87.7 ± 8.1	92.4 ± 8.5	0.001	72.7 ± 14.1	89.2 ± 9.7	<0.001
*V*_15_	72.6 ± 6.1	86.2 ± 7.3	<0.001	86.7 ± 11.9	93.3 ± 9.4	0.002	76.5 ± 8.6	85.7 ± 7.7	<0.001	60.3 ± 14.3	83.0 ± 12.5	<0.001
*V*_20_	62.4 ± 5.7	78.1 ± 8.4	<0.001	83.5 ± 12.1	91.1 ± 10.4	0.001	61.6 ± 9.3	75.8 ± 9.4	<0.001	48.9 ± 13.6	72.8 ± 14.2	<0.001
*V*_25_	51.4 ± 5.6	65.2 ± 7.9	<0.001	79.3 ± 12.0	87.4 ± 11.2	<0.001	46.1 ± 8.6	58.2 ± 10.0	<0.001	37.4 ± 12.0	58.3 ± 14.7	<0.001
*V*_30_	40.8 ± 5.3	51.8 ± 8.0	<0.001	72.6 ± 11.6	79.9 ± 11.8	0.001	33.0 ± 7.2	41.1 ± 8.5	<0.001	26.3 ± 9.7	44.1 ± 14.9	<0.001
*V*_35_	31.1 ± 5.3	38.1 ± 7.3	<0.001	61.5 ± 10.6	67.0 ± 11.5	0.010	22.6 ± 6.5	26.6 ± 7.3	0.003	18.0 ± 8.1	30.4 ± 12.3	<0.001
*V*_40_	22.8 ± 4.6	26.8 ± 6.2	<0.001	49.3 ± 9.2	52.9 ± 11.1	0.059	14.6 ± 4.9	15.9 ± 5.0	0.169	12.2 ± 6.1	20.1 ± 9.1	<0.001
*V*_45_	16.0 ± 3.8	18.0 ± 5.4	0.049	37.4 ± 8.0	38.5 ± 10.9	0.536	9.4 ± 3.5	9.7 ± 3.9	0.631	7.9 ± 4.4	12.3 ± 6.6	<0.001
*V*_50_	8.9 ± 3.0	8.9 ± 5.0	0.930	21.6 ± 6.5	20.7 ± 11.7	0.604	4.5 ± 2.3	4.4 ± 3.0	0.778	3.6 ± 2.9	5.3 ± 4.4	0.027
*D_mean_*	2635 ± 212	3057 ± 271	<0.001	3682 ± 575	3867 ± 430	0.060	2494 ± 263	2790 ± 267	<0.001	2126 ± 423	2817 ± 523	<0.001

### Comparison of Acute Adverse Events Between Two Groups

All patients completed planned radiotherapy with a median duration of 35 days (ranging from 31–52 days). In the PBM sparing IMRT group, 1 patient suspended radiotherapy due to perianal pain; 3 patients suspended chemotherapy due to severe diarrhea (1 case), loss of appetite (1 case) and HT (1 case); 3 cases experienced a decrease in chemotherapy dose due to vomiting and nausea (1 case) and mild diarrhea (2 cases). In the non-PBM sparing IMRT group, 5 patients suspended radiotherapy due to perianal pain (2 cases), perianal dermatitis (2 cases) and vomiting (1 case); 1 patient stopped chemotherapy halfway through treatment due to severe HT; 3 cases suspended chemotherapy due to diarrhea (2 cases) and nausea and perianal dermatitis (1 case).

All acute adverse events are shown in **Table 5** for both groups. For the acute HT observation, the numbers of the patients with grade 0, 1, 2, 3, 4 acute HT were 18(30.0%), 23(38.3%), 18(30.0%), 1(1.7%), and 0(0.0%) respectively in the PBM sparing IMRT group; while the corresponding numbers were 14(26.9%), 11(21.2%), 19(36.5%), 8(15.4%) and 0(0.0%) in the non-PBM sparing IMRT group. The incidence of acute HT (χ^2^ = 9.685, *P*=0.021), especially for the grade 3 or upper acute HT (χ^2^ = 7.094, *P*=0.008), was significantly reduced in patients treated with PBM sparing IMRT compared with patients treated with non-PBM sparing IMRT.

**Table 5:** Comparison of acute adverse events between PBM sparing IMRT group and non-PBM sparing IMRT group.

Adverse events	PBM sparing IMRT group				non-PBM sparing IMRT group				χ^2^	P
	Grad0	Grad1	Grad2	Grad3	Grad0	Grad1	Grad2	Grad3		
Hematologic toxicity	18	23	18	1	14	11	19	8	9.685	0.021
Vomiting	59	0	1	0	50	1	1	0	1.178	0.555
Diarrhea	46	8	5	1	34	7	5	6	4.892	0.18
Fatigue	59	1	0	0	51	1	0	0	0.01	0.919
Anorexia	55	4	1	0	46	5	1	0	0.343	0.842
Nausea	57	2	1	0	50	1	1	0	0.221	0.895
Hand-foot syndrome	59	1	0	0	51	1	0	0	0.01	0.919
Cystitis	60	0	0	0	50	2	0	0	2.35	0.125
Perianal pain	56	3	1	**0**	48	1	3	0	2.054	0.358
Perianal dermatitis	52	4	3	1	50	0	1	1	4.491	0.213

As for other adverse events, there was no statistical difference in vomiting, diarrhea, fatigue, anorexia, nausea, hand-foot syndrome, cystitis, perianal pain, and perianal dermatitis for both groups (*P*>0.05). Among those adverse events, the incidence of grade 2 or upper diarrhea was not high (10% V.S. 21.2%) in both groups. In addition, the occurrence probability of grade 2 or upper vomiting, anorexia, nausea, perianal pain was low (less than 2%) in both groups. There was no occurrence of grade 2 or upper fatigue, hand-foot syndrome and cystitis for both groups.

## Discussion

Acute HT is a common adverse event in rectal cancer patients undergoing CRT (5–8). Serious HT could lead to increasing risk of infection and an extended treatment period. Moreover, it could lead to delayed or missed chemotherapy cycles and treatment breaks, potentially compromising disease control (11). Multiple studies indicated that an increased radiation dose to PBM is associated with a higher incidence of acute HT in pelvic cancer patients undergoing CRT (21, 27). Hence, reducing the PBM radiation dose might reduce the incidence of acute HT in rectal cancer patients with CRT treatment. It is found that IMRT has great potential to spare PBM in pelvic malignancy because it can generate dose distributions that conform to the target and reduce the dose to the surrounding tissues (28).

To our knowledge, our study is the first to evaluate the clinical efficacy of PBM sparing in rectal cancer patients treated with concurrent IMRT and chemotherapy. There were several retrospective studies over the past few years regarding the correlation of PBM dose-volume parameters with the incidence of acute HT for patients with pelvic malignancy in IMRT radiotherapy procedure (9, 10, 15, 16, 21, 22). However, we have not found any report referring to CT image contoured PBM-sparing in rectal cancer patients.

A key unanswered question is: What is considered the best dose-volume PBM parameter and to what degree of PBM sparing can acute HT be reduced to permit better chemo-radiotherapy delivery in patients undergoing CRT. Some studies suggested that the V_10_, V_20_ and the D_mean_ of PBM were related to acute HT in patients with pelvic malignancy (15, 16, 21, 22), and PBM V_30_ was related to the occurrence of irreversible morphologic BM changes (13). Other studies demonstrated that lumbosacral spine BM’s V_40_ <60% (10) and V_45_ <51% (9) indicate lower probability of acute HT in rectal cancer patients. However, those constraints were relatively loose compared with that of our clinical practice. For example, lumbosacral spine BM’s V_40_ and V_45_ in our non-PBM sparing IMRT group were 52.9 ± 11.1% and 38.5 ± 10.9% which were much lower than the above mentioned recommendations. Hence, only the PBM V_10_, V_20_ and V_30_ were chosen to be spared in our study. It is noteworthy that the PBM D_mean_ was not restricted in our study because it would be simultaneously reduced along with the dosimetric constraints of PBM V_10_, V_20_ and V_30_. The PBM dosimetric constraints were carefully tuned to reduce the radiation dose to PBM in PBM sparing IMRT group. After trial and failure, the final dosimetric constraints were given as: V_10_ ≤ 85%, V_20_ ≤ 65% and V_30_ ≤ 45%.

In our study, we found that setting dosimetric constraints for PBM(V_10_ ≤ 85%, V_20_ ≤ 65% and V_30_ ≤ 45%) during IMRT plan optimization can significantly reduce most of the volume of PBM and sub-regions that received low and medium dose. The radiation dose to PBM (V_5_~V_45_, D_mean_, *P*<0.05), PBM sub-regions (V_10_~V_35_, D_mean_, *P*<0.05) and femoral heads (V_5_~V_40_, D_mean_, *P*<0.05) decreased significantly in the PBM sparing IMRT group compared with that of the non-PBM sparing IMRT group (*P*<0.05). In the meantime, this moderate degree of PBM sparing would neither sacrifice the dose coverage of the PTV (*P*>0.05) nor increase the radiation dose to bladder and small bowel (*P*>0.05). Thus, setting dose constraint V_10_ ≤ 85%, V_20_ ≤ 65% and V_30_ ≤ 45% to PBM was a feasible attempt to reduce the radiation dose to PBM.

The moderate degree of PBM sparing in our study could statistically reduce the incidence of acute HT (χ2 = 9.685, *P*=0.021), especially the grade 3 acute HT (1.7% V.S. 15.4%, *P*=0.008) in the PBM sparing IMRT group. No patients experienced a decrease in chemotherapy dose due to serious acute HT. PBM sparing could better guarantee the complete treatment of patients, thus improving the treatment efficiency compared with those patients who were treated with non-PBM sparing IMRT (1 patient stopped chemotherapy halfway through treatment due to severe HT in non-PBM sparing IMRT group).Moreover, there was no statistical difference in vomiting, diarrhea, fatigue, anorexia, nausea, hand-foot syndrome, cystitis, perianal pain, and perianal dermatitis in either groups (*P*>0.05). In short, all these findings suggest that the PBM constraints adopted in our study (V_10_ ≤ 85%, V_20_ ≤ 65% and V_30_ ≤ 45%) could achieve clinically significant reductions in acute HT while without increasing the incidence of other adverse events.

However, several limitations are noted in our study. First, we only enrolled rectal cancer patients in our department, which may have resulted in possible regional bias. Second, the VMAT technique, which has stronger dose modulation capabilities than fixed-field IMRT technique, was applied to a larger proportion of patients in PBM sparing IMRT group than those patients in the non-PBM sparing IMRT group because of the promotion of VMAT technique. Further studies are needed in order to compare the differences between the fixed-field IMRT technique and the VMAT technique. Third, we found that the dose constraint of small bowel in some patients could hardly meet the standard(V_45_ ≤ 195 cc) during IMRT plan optimization. There were two reasons: 1) due to our delineating method of small-bowel (outlining the entire potential space of small-bowel location), a certain volume of small bowel overlapped with the PTV. 2) The dose limitation of small bowel in our study was partially sacrificed to ensure e adequate dose coverage of the PTV (50Gy). Nevertheless, acute adverse events observation showed that patients in both groups experienced a low amount of grade 2 and upper diarrhea, and there was no statistical difference in diarrhea in both groups (10% V.S. 21.2%, *P*>0.05). Further research is needed for clarification.

## Conclusion

We attempted to investigate the feasible dosimetric constraints for PBM that can be widely used in rectal cancer patients treated with concurrent IMRT and chemotherapy. Applying PBM dosimetric constraints (V_10_ ≤ 85%, V_20_ ≤ 65% and V_30_ ≤ 45%) could effectively reduce the radiation dose to PBM, thus significantly reducing the incidence of acute HT, while without increasing the probability of other adverse events. The PBM sparing standard proposed in our study provides a more practical guideline to reduce the incidence of acute HT for rectal cancer patients. This new standard may benefit patients in improving the treatment efficacy for rectal cancer patients undergoing CRT.

## Data Availability Statement

The original contributions presented in the study are included in the article/**Supplementary Material**, further inquiries can be directed to the corresponding author.

## Ethics Statement

The studies involving human participants were reviewed and approved by Ethical and Scientific Committees of The First Affiliated Hospital of Chongqing Medical University (#2020-677, Chongqing, China). The patients/participants provided their written informed consent to participate in this study.

## Author Contributions

WH, YL and Q-fJ drafting of work, analysis and interpretation of trials and literature, drafting of manuscript, and manuscript review. WH, H-xC, W-lL collected the data, reviewed the literature, and wrote the paper. JD and Q-fJ prepared the figure and contributed in the revision of the literature. All authors contributed to the article and approved the submitted version.

## Conflict of Interest

The authors declare that the research was conducted in the absence of any commercial or financial relationships that could be construed as a potential conflict of interest.
