# Supplementary figures and images for: Effect of Pelvic Bone Marrow Sparing Intensity Modulated Radiation Therapy on Acute Hematologic Toxicity in Rectal Cancer Patients Undergoing Chemo-Radiotherapy
Source: Front Oncol. 2021 Apr 22;11:646211. doi: 10.3389/fonc.2021.646211 (PMC8101329; doi:10.3389/fonc.2021.646211)

## Slide 1
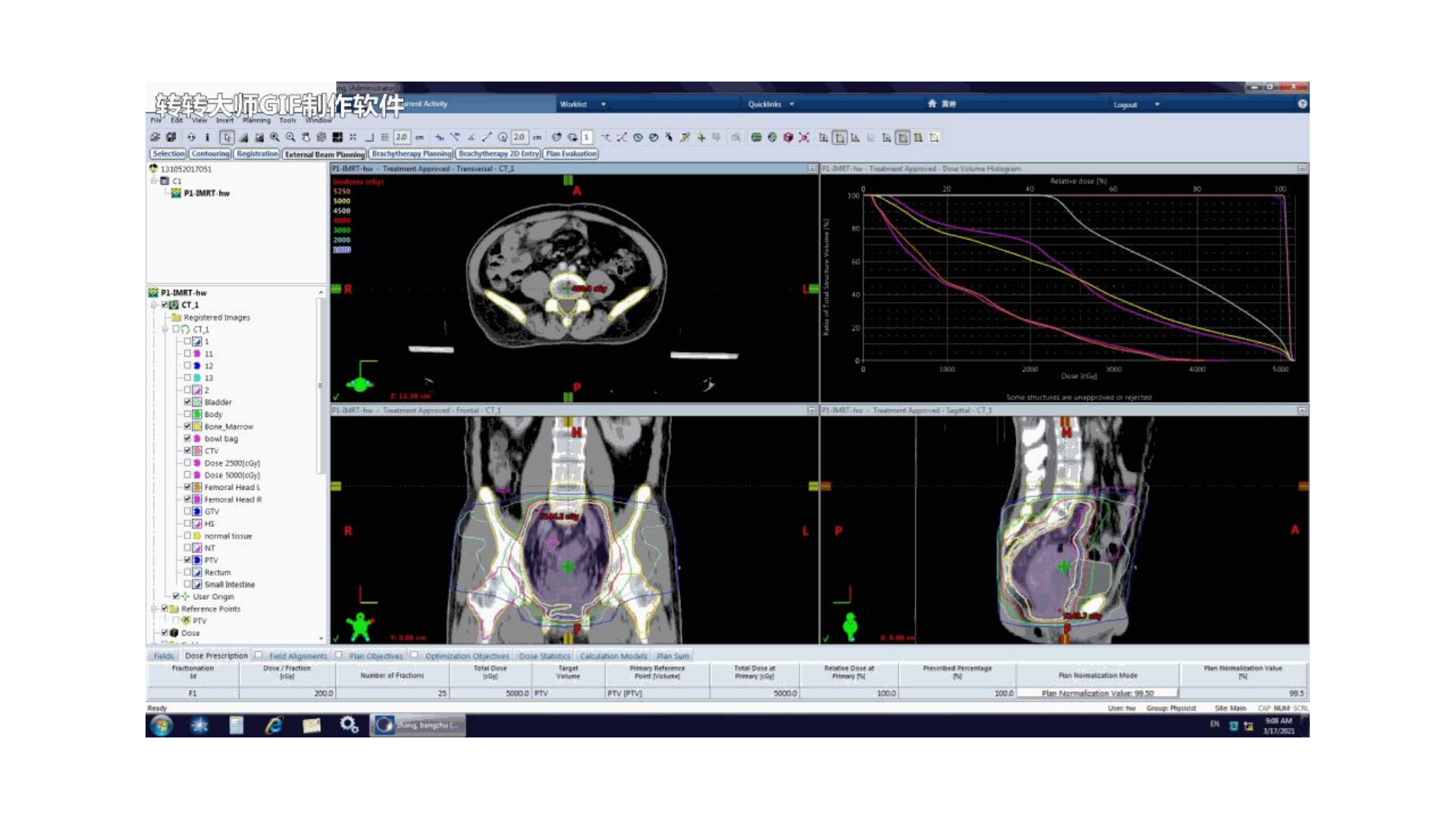

Supplement: Supplementary file 1 [file Presentation_1.pptx]
